# Prokaryotic communities profile from metagenomic libraries of the brown rock sea cucumber (*Holothuria glaberrima*) intestinal system

**DOI:** 10.1128/mra.00731-25

**Published:** 2025-08-25

**Authors:** Rene Nieves-Morales, Jessica Alejandra Paez-Diaz, Edwin Omar Rivera-Lopez, Josué Rodríguez-Ramos, Carlos Rios-Velazquez

**Affiliations:** 1 Microbial Biotechnology and Bioprospecting Laboratory, Biology Department, University of Puerto Rico at Mayagüez 585475, Mayagüez, Puerto Rico, USA; 2 Department of Food Science, The Pennsylvania State University 730256, University Park, Pennsylvania, USA; 3 Biological Sciences Division, Pacific Northwest National Laboratory 536904 , Richland, Washington, USA; Montana State University, Bozeman, Montana, USA

**Keywords:** 16S, metagenomic libraries, sea cucumber, *Holothuria glaberrima*, amplicon

## Abstract

Environmental rDNA profiling enables the identification of unculturable microbial communities. To access prokaryotic diversity in the metagenomic libraries of sea cucumber’s intestinal environment, 16S rDNA sequencing was performed to provide insight into the libraries’ taxonomic composition, unraveling microbial groups potentially associated with biomedical, environmental, and biotechnological applications.

## ANNOUNCEMENT

Amplicon sequencing is a powerful tool used to uncover unculturable environmental microbial communities (1). The 16S rDNA amplicons are a common tool for prokaryotic microbiomes as they offer advantages due to their extensive reference database (2). This analysis approach offers insights into taxonomic composition and possible metabolic roles attached to the microbial diversity represented in metagenomic libraries (3, 4). The regeneration of the gastrointestinal system in the sea cucumber (*Holothuria glaberrima*) may be influenced by prokaryotic communities that contribute to digestive functions through enzymes such as proteases, lipases, and cellulases. Particularly, cellulase genes, absent from the host genome but produced by the genera in the sea cucumber’s gut, including *Clostridium*, *Ruminococcus*, *Cellvibrio*, *Bacillus*, and *Pseudomonas* (5). Also, further studies on the exploration of marine microbial enzymes in the biotechnology industry might aid in the discovery of novel products (6, 7). Previous studies have shown that antibiotics negatively impact regenerative and digestive functions, highlighting the microbiome’s importance in physiological functions (5, 8).

Thirteen *H. glaberrima* specimens were collected from Piñones Beach, San Juan (18.451141, −65.905634) and maintained in a marine water aquarium for acclimatization (4, 5). Samples were divided as follows: washed intestine (WI), contents from the washed intestine (CW), fecal content (FC), and digestive system (DS). Direct metagenomic DNA extraction combining mechanical (freeze and thaw), enzymatic (lysozyme), and chemical (sodium dodecyl sulfate and guanidinium thiocyanate) methods was employed. Additionally, library generation and fosmid extraction (pCC1Fos) were performed as described in the literature (4, 9). A total of 267,405 clones were generated and distributed as follows: FC (1,107), CW (56,300), WI (208,615), and DS (1,320). Metagenomic DNA quality was assessed with a NanoDrop (Thermo Scientific NanoDrop Products) (10, 11) and sent for short-read Illumina 16S rDNA amplicon sequencing to Molecular Research Laboratory, MR. DNA (https://www.mrdnalab.com/), using F (CCTACGGGNGGCWGCAG) and R (GACTACHVGGGTATCTAATCC) primers for variable regions V3–V4 (12, 13). Samples were fragmented, adapter sequences added, and sequencing performed on the Illumina MiSeq system (2 × 300 bp paired-end reads in 30 cycles) with MiSeq Reagent Kit v3 (14), resulting in 285,085 reads of taxa data and processed using QIIME-2 (amplicon-2024.5) (15) with default parameters, except noted otherwise. Cutadapt (v.4.9) was used to remove primers (16), and DADA2 (1.24.0) was used for denoising with parameters set at “-p-trunc-len-f 215” and “-p-trunc-len-r 225” (17), resulting in 233,370 quality reads. The data were clustered into 46 amplicon sequence variants at a 100% similarity threshold, with 66.56% of denoised sequences being non-chimeric. Taxonomy was assigned with q2-feature-classifier (18), classify-sklearn (v.1.4.2) (19), and SILVA database (classifiers/silva-138-99-nb-classifier.qza) (20). Bar plot generation was generated in RStudio (4.4.2) using the packages as follows: ggplot2, dplyr, tidyr, scales, randomcoloR, and grid (21).

**Fig 1:**
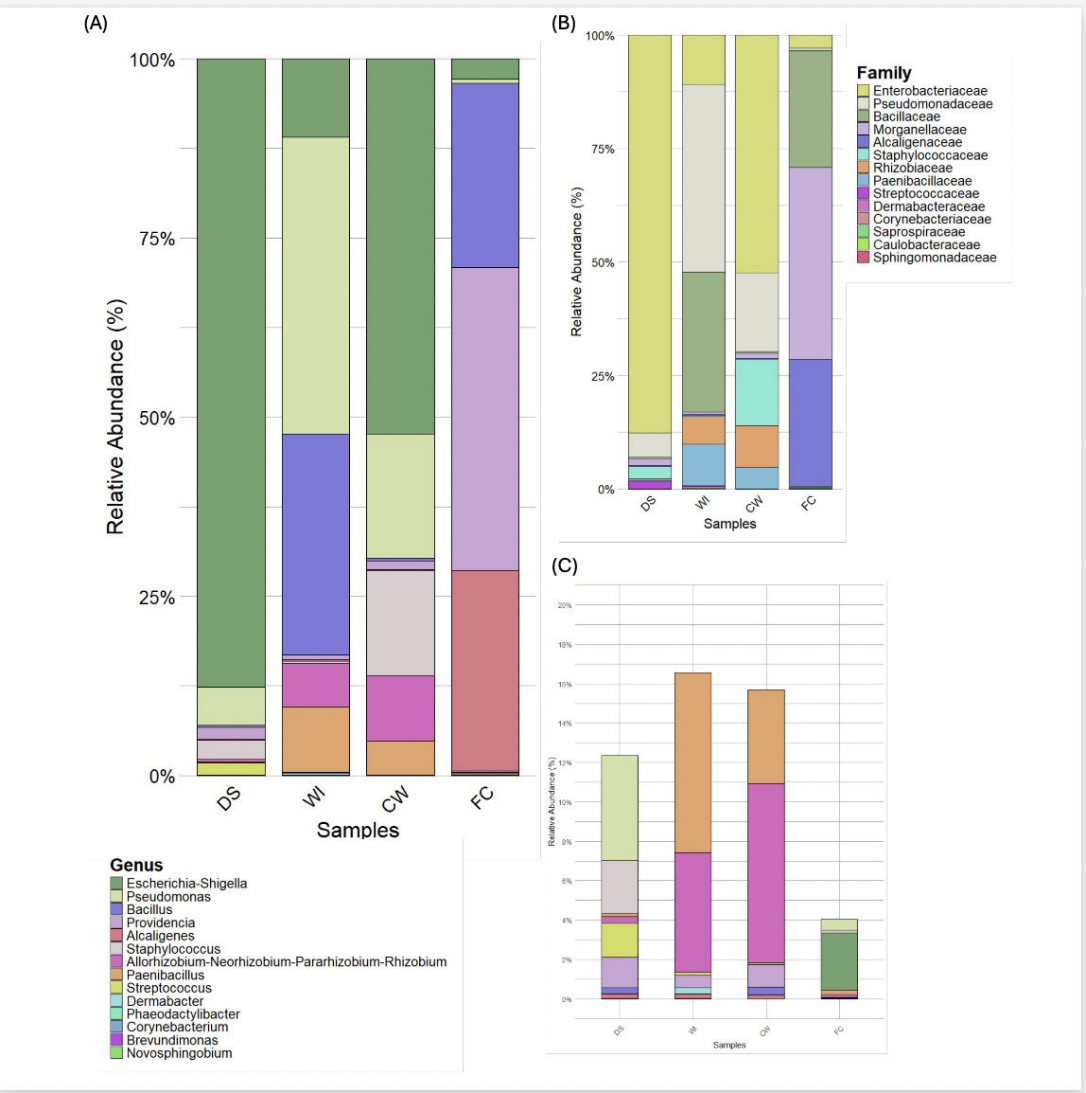
Prokaryotic diversity of the gastrointestinal system of *H. glaberrima* across different sections of the DS. The complete DS, WI, CW, and FC. (**A**) Taxa bar plot displays the relative abundance at the genus level, (**B**) shows the relative abundance at the family level, and (**C**) shows the low abundance levels at the genus level.

Fourteen bacterial families and genera were identified (Fig. 1A thorugh C). DS was dominated by *Enterobacteriaceae* (86.829%) and *Pseudomonadaceae* (5.296%), corresponding to *Escherichia*–*Shigella* (86.829%) and *Pseudomonas* (5.296%). CW was characterized by *Enterobacteriaceae* (52.011%) and *Rhizobiaceae* (9.024%), which aligned with *Escherichia*–*Shigella* (52.011%) and *Staphylococcus* (14.556%). WI showed predominance of *Pseudomonadaceae* (41.274%) and *Bacillaceae* (30.657%), corresponding to *Pseudomonas* (41.274%) and *Bacillus* (30.657%), respectively. FC was primarily composed of *Morganellaceae* (42.156%) and *Alcaligenaceae* (27.970%), corresponding to *Providencia* (42.156%) and *Alcaligenes* (27.970%).

## Data Availability

The raw data of this project has been deposited in the National Center for Biotechnology Information (NCBI), under the BioProject accession number PRJNA1199568. The sample barcodes and SRA accession numbers are the following: *H. glaberrima*: complete digestive system—DS (barcode CAGTTCAT; SRA SRS23572270); *H. glaberrima*: washed intestine—WI (barcode CATAATAG; SRA SRS23572271); *H. glaberrima*: contents from the washed intestine—CW (barcode CAGTTGCA; SRA SRS23572272); *H. glaberrima*: fecal content—FC (barcode CATAACAA; SRA SRS23572273).
